# Splign: algorithms for computing spliced alignments with identification of paralogs

**DOI:** 10.1186/1745-6150-3-20

**Published:** 2008-05-21

**Authors:** Yuri Kapustin, Alexander Souvorov, Tatiana Tatusova, David Lipman

**Affiliations:** 1 National Center for Biotechnology Information, National Library of Medicine, National Institutes of Health, Bethesda, MD 20814, USA

## Abstract

**Background:**

The computation of accurate alignments of cDNA sequences against a genome is at the foundation of modern genome annotation pipelines. Several factors such as presence of paralogs, small exons, non-consensus splice signals, sequencing errors and polymorphic sites pose recognized difficulties to existing spliced alignment algorithms.

**Results:**

We describe a set of algorithms behind a tool called Splign for computing cDNA-to-Genome alignments. The algorithms include a high-performance preliminary alignment, a compartment identification based on a formally defined model of adjacent duplicated regions, and a refined sequence alignment. In a series of tests, Splign has produced more accurate results than other tools commonly used to compute spliced alignments, in a reasonable amount of time.

**Conclusion:**

Splign's ability to deal with various issues complicating the spliced alignment problem makes it a helpful tool in eukaryotic genome annotation processes and alternative splicing studies. Its performance is enough to align the largest currently available pools of cDNA data such as the human EST set on a moderate-sized computing cluster in a matter of hours. The duplications identification (compartmentization) algorithm can be used independently in other areas such as the study of pseudogenes.

**Reviewers:**

This article was reviewed by: Steven Salzberg, Arcady Mushegian and Andrey Mironov (nominated by Mikhail Gelfand).

## Background

Spliced gene products available in the form of cDNA sequences provide an experimental level of support for gene models. It has been shown [1] that the availability of large numbers of such sequences greatly improves the quality of identification of gene structures, especially in UTR regions which are beyond the application scope of most *ab initio *gene-prediction methods. Accuracy of spliced alignments is crucial in such areas as studies of alternative splicing and regulatory elements.

Over the last decade, significant attention has been given to development of tools to assist the spliced alignment problem. A useful overview of such tools has been given in [2]. Despite considerable progress in more recent tools, various types of alignment errors are still observed. Such errors include missing micro-exons, forced consensus splice signals and alignments stretching over several members of tandem gene clusters. Another critical issue is the performance of the algorithms.

**Figure 1:**
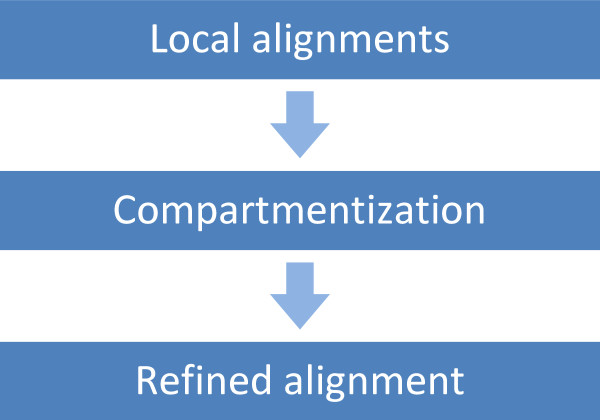
The computation of spliced alignments with Splign.

We developed a tool called Splign for accurate and fast alignment of spliced cDNA sequences against their genomic counterparts. The process (Figure 1) starts with computing local alignments between the input cDNA set and the genome. The local alignments are used to identify candidate locations on the genome for every cDNA. Every location is then refined using an optimal alignment algorithm specifically accounting for possible splice sites.

This general scheme has been exemplified in virtually every spliced alignment method capable of aligning a cDNA against a whole genomic assembly. Our approach is different in that it uses a formally defined model of same-strand duplications, which are found as a solution of an optimization problem. Splign is very conservative in its use of local alignments to seed the core splice refinement algorithm, which tends not to bind the final alignment with preliminary alignments delivering non-unique mappings. This also makes the algorithm more capable of finding small exons which are often missed by other methods. Finally, we explicitly list several important alignment alternatives and assign the elementary scores of the optimal alignment algorithm via a system of inequalities assuring preferable alignment outcomes.

## Results and Discussion

To assess the quality of alignments reported by Splign, we compared it with five other spliced alignment programs: Sim4 [3], Spidey [4], BLAT [5], GMAP [2], and SPA [6] (Table 1). We used each of the programs to produce alignments of 218 641 human mRNA sequences with the reference human genome (build 36.3). The alignments were then compared using different quality measures. In a separate test, we also aligned 1 683 827 EST sequences that were expected to have same splicing forms as selected RefSeq [7] mRNA sequences. The EST alignments were then compared to the alignments of the corresponding RefSeq mRNA sequences.

**Table 1:** Programs used in the comparison

	Sim4	Spidey	BLAT	GMAP	SPA	Splign
version	-	1.40	34	2007-06-04	-	1.29
source time stamp	09/2003	06/2006	04/2007	06/2007	07/2007	08/2007

### Identity-based comparison

Full-length mRNA sequences are high-quality transcripts each representing a splicing variant of a gene. To assess the quality of alignment of a cDNA against a genomic locus, we introduced the following measures. *Overall identity *is the number of matching residues, divided by length of the alignment excluding possible introns. For the purpose of this definition, cDNA bases that failed to align (except those of the poly(A) tail, if any) are counted as deletions. For example, if 62 out of the total of 100 bases of a cDNA aligned perfectly and the other 38 bases did not align, the overall identity is 62%.

If information about the coding region is available, it is possible to introduce a measure accounting for frame shifts. Frame shifts in a coding region's alignment are caused by gaps whose length is not a multiple of three, and usually indicate either an error in a cDNA or genomic sequence, or incorrect alignment. *In-frame identity *is the number of matching nucleotide residues aligned without a frame shift, divided by the length of the coding region's alignment excluding possible introns.

The presence of same-strand duplications often poses a problem for spliced alignment algorithms. With the possibility of a sequencing error or a polymorphic site on the genome, the alignment with the highest identity can stretch across multiple duplicated regions. Compactness of an alignment can be quantified with its *span ratio*, which is the span of the alignment on the genome divided by the length of the cDNA sequence.

Throughout our tests, the genome was represented as a collection of chromosomes and unplaced scaffolds. Four of the programs (BLAT, GMAP, SPA and Splign) are able to align a cDNA against the whole genome. Sim4 and Spidey require externally specified genomic sequence. We found that running these two programs on full-length human chromosomes will often cause them to crash. Assuming that in practice users would most likely run these two programs against genomic scaffolds, for every cDNA we supplied Sim4 and Spidey with scaffolds where Splign reported at least partial alignment.

Our set of mRNA sequences consisted of 218 641 human mRNA sequences available at GenBank at the time of the testing, with 24 273 of them being RefSeq mRNA sequences.

After having computed the alignments, we found that it was not uncommon for the programs to report 3' exons consisting mostly or entirely of 'A' residues, often connected to the rest of the gene by a non-consensus intron (we call an intron consensus if it has one of the following donor/acceptor pairs: GT/AG, GC/AG, AT/AC). We suspect that in many cases such alignment segments are in fact poly(A) tails that should not have been aligned. Table 2 lists the numbers of mRNA sequences with alignments featuring A-content of 75% or higher in their 3' segments. The data shows that the approaches for recognizing and trimming possible poly(A) tails lead to varying results among the programs under comparison. When computing the alignment statistics the maximum tail of consecutive 'A' residues was checked to allow one or two non-'A' residues. If such a tail was found, the alignment was trimmed to the start of the first all-'A' substring of length five or longer. Any alignment beyond that point was then ignored.

**Table 2:** The number of best alignments with A-content of 75% or higher in the 3' exon. The numbers are based on the alignments with the highest overall identity.

	Sim4	Spidey	BLAT	GMAP	SPA	Splign
%% of all mRNAs	15.5	0.57	0.98	0.04	2.12	0.01
%% of RefSeq mRNAs	22.1	0.31	1.67	0.02	2.72	0.00

Tables 3 and 4 compares the number of sequences aligned at various levels of the overall identity by Splign versus the other tools. The data shows that at the higher identity levels Splign was able to align more sequences than any other tool. Table 5 lists the total time it took for every program to compute the alignments for the full set of mRNA sequences.

**Table 3:** The number of the full set alignments at various levels of the overall identity. Fractions give the differences in the number of alignments by each method and Splign expressed as percentage of the total of sequences in the set.

	80%	85%	90%	95%	99%	99.5%	99.9%	100%
Splign	212875	209895	203460	195939	182123	169058	107267	76542
Sim4	212533	208571	203016	195513	177784	162761	97812	69359
	-0.16	-0.61	-0.20	-0.19	-1.98	-2.88	-4.32	-3.29
Spidey	199870	195159	190753	186356	172385	159021	100338	72225
	-5.95	-6.74	-5.81	-4.38	-4.45	-4.59	-3.17	-1.97
BLAT	209899	206260	201207	194640	177345	163157	104338	74815
	-1.36	-1.66	-1.03	-0.59	-2.19	-2.70	-1.34	-0.79
GMAP	208849	205793	202732	196734	180048	166488	105452	74975
	-1.84	-1.88	-0.33	+0.36	-0.95	-1.18	-0.83	-0.72
SPA	203942	199548	195926	192595	180273	167815	105835	74911
	-4.09	-4.73	-3.45	-1.53	-0.85	-0.57	-0.65	-0.75

**Table 4:** The number of RefSeq alignments at various levels of the overall identity. Fractions give the differences in the number of alignments by each method and Splign expressed as percentage of the total of sequences in the set.

	80%	85%	90%	95%	99%	99.5%	99.9%	100%
Splign	24255	24254	24243	24218	23947	23420	19337	14898
Sim4	24242	24233	24201	24109	23566	22815	17774	12894
	-0.05	-0.09	-0.17	-0.45	-1.57	-2.49	-6.44	-8.26
Spidey	23648	23491	23270	22972	22408	21789	17782	13488
	-2.50	-3.14	-4.01	-5.13	-6.34	-6.72	-6.41	-5.81
BLAT	24240	24230	24201	24145	23701	23046	19043	14676
	-0.06	-0.10	-0.17	-0.30	-1.01	-1.54	-1.21	-0.91
GMAP	24249	24242	24225	24198	23876	23294	19217	14513
	-0.02	-0.05	-0.07	-0.08	-0.29	-0.52	-0.49	-1.59
SPA	24213	24202	24180	24148	23830	23303	19204	14698
	-0.17	-0.21	-0.26	-0.29	-0.48	-0.48	-0.55	-0.82

**Table 5:** Time to compute alignments for the full set of human mRNA sequences. The timing is based on a single instance running on Intel Xeon 2.33 GHz/8 GB Linux box.

	Sim4	Spidey	BLAT	GMAP	SPA	Splign
CPU hours	856	698	8	12	2448	49

Although the full set of mRNA sequences is the most representative, one may argue that the comparison based on it could be biased. A fraction of mRNA sequences are deposited to GenBank as complemented strand. Splign can report both sense and anti-sense alignments for a single mRNA which may give it an advantage over the tools that report alignments in one direction, because an incorrectly directed alignment can have an identity higher than the one in the correct direction. For queries aligning to more than one place on the genome, strategies vary among the tools, with some reporting all alignments above certain quality threshold and others attempting to rank the alignments and report a fixed number of the top-ranking alignments. To minimize these differences, we restricted the full set of mRNAs using the following conditions:

• single alignment with 80% or higher overall identity

• sense maximal ORF is 900 bases or longer

• anti-sense maximal ORF is at least two times smaller

The conditions produced a subset consisting of 72 113 mRNA sequences and 13 883 RefSeq mRNA sequences ("Subset 1").

Tables 6 and 7 present the comparison data based on the Subset 1 alignments. The data shows that at every identity level Splign was able to align more sequences than any other tool, most closely followed by SPA and GMAP.

**Table 6:** The number of the Subset 1 alignments at various levels of the overall identity. Fractions give the differences in the number of alignments by each method and Splign expressed as percentage of the total of sequences in the set.

	85%	90%	95%	99%	99.5%	99.9%	100%
Splign	72047	71922	71568	68890	65666	43046	25931
Sim4	72013	71830	71316	68055	63919	39272	23249
	-0.05	-0.13	-0.35	-1.16	-2.42	-5.23	-3.72
Spidey	71562	70833	69744	66128	62461	40456	24578
	-0.67	-1.51	-2.53	-3.83	-4.44	-3.59	-1.88
BLAT	72010	71842	71393	67508	63319	41693	25357
	-0.05	-0.11	-0.24	-1.92	-3.25	-1.88	-0.80
GMAP	72001	71843	71459	68446	64787	42263	25335
	-0.06	-0.11	-0.15	-0.62	-1.22	-1.09	-0.83
SPA	71993	71823	71406	68578	65368	43043	25736
	-0.07	-0.14	-0.22	-0.43	-0.41	0.00	-0.27

**Table 7:** The number of the Subset 1 RefSeq alignments at various levels of the overall identity. Fractions give the differences in the number of alignments by each method and Splign expressed as percentage of the total of sequences in the set.

	85%	90%	95%	99%	99.5%	99.9%	100%
Splign	13883	13880	13870	13781	13540	11345	8276
Sim4	13880	13873	13832	13615	13287	10521	7151
	-0.02	-0.05	-0.27	-1.20	-1.82	-5.94	-8.10
Spidey	13765	13602	13379	13085	12802	10601	7587
	-0.85	-2.00	-3.54	-5.01	-5.32	-5.36	-4.96
BLAT	13878	13866	13843	13680	13355	11180	8167
	-0.04	-0.10	-0.19	-0.73	-1.33	-1.19	-0.79
GMAP	13879	13875	13864	13753	13481	11291	8066
	-0.03	-0.04	-0.04	-0.20	-0.42	-0.39	-1.51
SPA	13878	13868	13855	13738	13502	11290	8174
	-0.04	-0.09	-0.11	-0.31	-0.27	-0.40	-0.73

The data in Tables 8 and 9 use in-frame identity to compare the alignments produced by the methods for the Subset 1. The data shows that at every level of identity, Splign was able to align more sequences than the other tools. To eliminate a possible concern that the higher in-frame identity demonstrated by Splign alignments may be a result of excessive preference for non-consensus splices, we also counted the numbers of every splice type found in the alignments produced by each of the programs (Table 10). The comparison reveals that Splign non-consensus splice frequency is the second lowest, and the consensus splice counts are very close to those produced by the two other recent tools.

**Table 8:** The number of the Subset 1 alignments at various levels of the in-frame identity. Fractions give the differences in the number of alignments by each method and Splign expressed as percentage of the total of sequences in the set.

	80%	85%	90%	95%	99%	99.5%	99.9%	100%
Splign	67342	67241	67105	66766	65767	64938	50968	35839
Sim4	63968	63723	63465	62986	61668	60622	47820	34033
	-4.68	-4.88	-5.05	-5.24	-5.68	-5.99	-4.37	-2.50
Spidey	65276	65182	65066	64766	63844	62867	48714	34451
	-2.86	-2.86	-2.83	-2.77	-2.67	-2.87	-3.13	-1.92
BLAT	64017	63932	63817	63545	62747	62115	49946	35632
	-4.61	-4.59	-4.56	-4.47	-4.19	-3.91	-1.42	-0.29
GMAP	66568	66463	66317	65956	64856	64058	50589	35680
	-1.07	-1.08	-1.09	-1.12	-1.26	-1.22	-0.53	-0.22
SPA	66777	66669	66516	66152	65042	64186	50669	35679
	-0.78	-0.79	-0.82	-0.85	-1.01	-1.04	-0.41	-0.22

**Table 9:** The number of the Subset 1 RefSeq alignments at various levels of the in-frame identity. Fractions give the differences in the number of alignments by each method and Splign expressed as percentage of the total of sequences in the set.

	80%	85%	90%	95%	99%	99.5%	99.9%	100%
Splign	13757	13747	13740	13733	13723	13688	12426	10323
Sim4	13214	13179	13145	13110	13044	12958	11662	9680
	-3.91	-4.09	-4.29	-4.49	-4.89	-5.26	-5.50	-4.63
Spidey	13185	13176	13169	13162	13146	13089	11720	9706
	-4.12	-4.11	-4.11	-4.11	-4.16	-4.31	-5.09	-4.44
BLAT	13507	13498	13491	13484	13473	13441	12271	10284
	-1.80	-1.79	-1.79	-1.79	-1.80	-1.78	-1.12	-0.28
GMAP	13744	13733	13726	13718	13706	13671	12408	10307
	-0.09	-0.10	-0.10	-0.11	-0.12	-0.12	-0.13	-0.12
SPA	13715	13706	13698	13691	13676	13635	12373	10275
	-0.30	-0.30	-0.30	-0.30	-0.34	-0.38	-0.38	-0.35

**Table 10:** Frequencies of splice sites in Subset 1 alignments

	GT/AG	GC/AG	AT/AC	non-consensus
Sim4	96.21	0.78	0.06	2.96
Spidey	95.72	0.67	0.09	3.52
BLAT	97.87	0.74	0.10	1.29
GMAP	98.74	0.75	0.12	0.38
SPA	98.52	0.74	0.11	0.62
Splign	98.66	0.75	0.11	0.48

### Compartment test

An acknowledged difficulty for a spliced alignment tool is to properly localize an alignment in presence of nearby same-strand duplications. In order to test how well each tool handles the task, we created a set of mRNA sequences with each sequence covered at least 1.5 times by same strand Megablast hits to the same subject (a chromosome or an unplaced scaffold), and the highest-identity alignment subject to the following conditions.

• same exon count among the methods

• sense direction

• at most one non-consensus splice

• the identity is 90% or higher

The conditions produced 9383 mRNA sequences ("Subset 2"). For every mRNA sequence in the set, its highest-identity alignment's span ratio has been compared among the methods. Table 11 shows that Splign has the smallest mean ratio and the second smallest median ratio. As with the identity-based tests, trailing 'A' residues that were part of alignments were trimmed prior to computing the statistics. Had this not been done, the ratios for the methods with higher fraction of alignments retaining Poly(A) tails would have gone up.

**Table 11:** Span ratios of Subset 2 alignments

	Sim4	Spidey	BLAT	GMAP	SPA	Splign
median	4.179	4.201	4.234	4.378	4.218	4.190
mean	11.242	8.534	9.134	10.102	8.671	8.420

### Co-aligning EST test

Alignment of EST sequences is often more difficult due to shorter sequence length and higher error rates. Yet for most organisms, the bulk of transcript evidence comes in the form of ESTs as they are less expensive to produce in a high-throughput manner than full-length mRNA sequences. Therefore, it is important for a spliced alignment program to be able to compute accurate alignments of cDNA sequences with higher sequencing error rates such as in ESTs. To measure how well different programs cope with the task, the following test has been conducted.

We selected a subset of RefSeq mRNA sequences that align uniquely across the genome with an identity of 99% or higher, having at least two exons, and at least one co-aligning EST, which yielded 13975 sequences. For each sequence from this set, a list of EST sequences was compiled whose EST-to-mRNA alignment suggested the same splicing form. This selection was done by running Megablast [8] on query EST sequences against a database of the mRNA sequences and selecting ESTs with the number of unaligned bases less than ten, the maximum gap length less than four, and the overall alignment identity of 95% or higher. In such a way, the total of 1 683 827 ESTs were selected. Initially, we estimated using the EST-to-mRNA and (method-specific) mRNA-to-genomic alignments the number of introns expected in the EST-to-genomic alignments. Then every EST from the list was aligned on the genome using each of the methods, and the number of introns exactly matching those found in the mRNA alignments was collected.

The results of this test are presented in Table 12. The data shows that in terms of sensitivity, Splign produced a higher number than any other tool except SPA, whose fraction of identified introns was higher by 1.4%. However, the time it took to compute the EST alignments with Splign (37 CPU hours) was nearly twenty times smaller than that of SPA. The best specificity was demonstrated by GMAP with almost 99.5% of introns matching those found in the mRNA alignments, followed by Splign that correctly aligned 98.9% of introns. Sim4, which is one of the oldest programs, also demonstrated good specificity.

**Table 12:** Co-aligning EST test. 'Implied' is the number of introns estimated from EST-to-mRNA and mRNA-to-genomic alignments. 'Identified' is the number of introns found in EST-to-genomic alignments. 'Matching' is the number of introns in EST-to-genomic alignment matching those found in the mRNA-to-genomic alignments.

	Sim4	Spidey	BLAT	GMAP	SPA	Splign
Implied	3472173	3419307	3417203	3419975	3425734	3419138
Identified, as %% of Implied	3174725	3249426	3150040	3219665	3407890	3352628
	91.4	95.0	92.2	94.1	99.5	98.1
Matching, as %% of Identified	3128325	2813461	2810059	3202121	3294548	3314074
	98.5	86.6	89.2	99.5	96.7	98.9

Although each mRNA sequence in this initial test was required to have a high-identity alignment, for a number of sequences different methods produced different alignments. To reduce the possibility that the initial EST test might have been affected by the alignment errors introduced by the methods in their mRNA alignments and repeated in the EST alignments, we repeated the test with an extra requirement that the set of introns must be the same in the mRNA alignments produced by every method. This brought down the number of mRNA sequences to 7 923, and the number of EST sequences to 915 111. The results of the test are presented in the second line of Table 13, and are in line with the results from the initial test.

**Table 13:** Co-aligning EST test, based on mRNA-to-genomic alignments matching across the methods

	Sim4	Spidey	BLAT	GMAP	SPA	Splign
Implied	1784663	1784663	1784663	1784663	1784663	1784663
Identified, as %% of Implied	1652062	1691136	1642759	1679932	1771713	1745535
	92.6	94.8	92.0	94.1	99.3	97.8
Matching, as %% of Identified	1635634	1477759	1475183	1672383	1719549	1729834
	99.0	87.4	89.8	99.6	97.1	99.1

## Conclusion

We developed a tool that is robust enough to produce accurate cDNA-to-genomic alignments in a matter of hours on a moderate-sized computing cluster for the largest available cDNA data volumes such as the human or mouse EST libraries. Splign has a powerful compartmentization algorithm to identify and separate nearby same-strand duplications. The program is tolerant to sequencing errors and polymorphic sites due to its use of the true optimal alignment algorithm and a conservative application of the preliminary local alignments.

There are three aspects that are novel in Splign compared to other methods. First, we introduce a high-performance method using index-to-index comparison for computing preliminary local alignments. Second, a formally defined model of compartments discriminating between gene and exon duplication events is used to localize candidate genomic regions for every input cDNA. Finally, the scores used in the splice-aware optimal alignment algorithm are obtained as a solution of a linear programming problem reflecting selected types of target alignments. Although the resulting affine gap scoring model employed in Splign is less generic than probability-based scoring models such as [6], it allows the computation of alignments of comparable quality faster by an order of magnitude.

Splign has been evolving over the past five years. It is routinely used at NCBI to facilitate annotation of eukaryotic genomes.

The Splign web site [9] provides access to the source code in C++ and allows the download of pre-compiled Splign and Compart binaries for several major platforms. The site also has a job submission facility, where cDNA queries can be aligned online against a genomic sequence or a whole genome.

## Methods

### Preliminary sequence alignment

**Figure 2:**
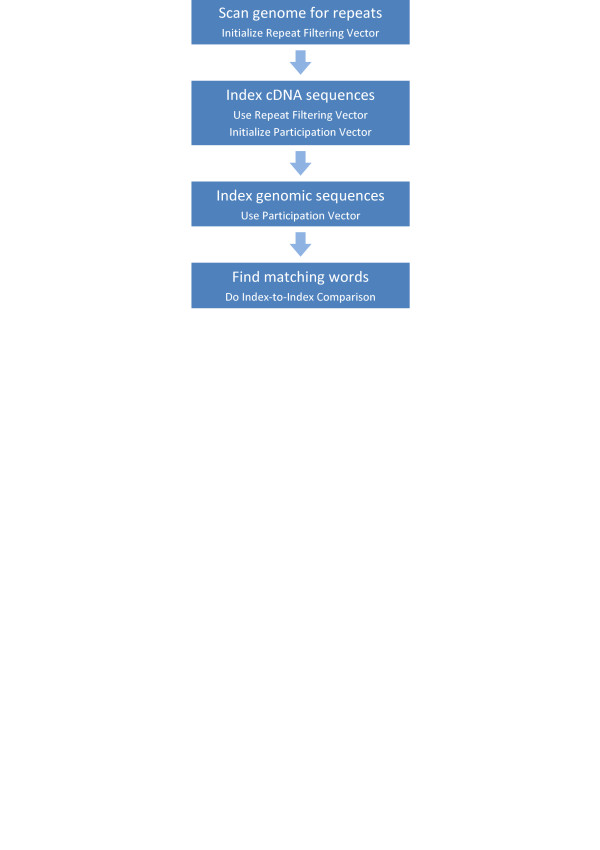
Compart matching algorithm.

In this section we describe the algorithm for the computation of elementary alignments between a set of input cDNA sequences and a genome from the same species (Figure 2). The goal was to make the algorithm both sensitive and fast when matching a large number of cDNA sequences against a whole genome.

High sensitivity of the algorithm is achieved by using a small word size and very light repeat filtering. During the compartmentization each alignment is evaluated in the context of compartments and kept only if found to be a member of the globally optimal chain of alignments. If some minimum level of query cDNA coverage by a compartment is employed, which is typical in practical applications, most spurious matches between a pair of sequences are discarded even before the compartmentization stage.

On the performance side, the algorithm benefits from utilizing the information about the composition of the cDNA sequences at the indexing of the genome, reducing the size of the index. Indices produced by the algorithm are stored in disk files to free memory for accumulation and processing of matching words. The latter are found by a linear-time comparison of the cDNA and genomic indices, during which the indices are accessed sequentially. The algorithm performs ungapped extension of the alignments as it is sufficient for the compartmentization and gaps in the final alignments are discovered using the target function of the refinement stage.

The algorithm starts with scanning the genomic sequence for repeats. Note that application of some form of repeat masking is necessary to keep a local alignment tool of choice from being overwhelmed with hits to repetitive genomic segments. On the other hand, accuracy of solutions produced by the compartmentization may suffer if the set of local alignment is incomplete. Since it is possible for a repeated sequence to be part of an exon, we apply a very light repeat filtering based on frequency counts of sparse words. We first collect the counts of 14-mers with relative positions 1, 2, 5 – 16 in 16-mers starting at every fourth position in the genome. Then we set elements of a repeat filtering bit vector (RFV) corresponding to 14-mers within the 99.5 percentile. For every 16-base word parsed during the cDNA indexing, the 14-base subsequence of the word is extracted and used to check the corresponding bit in the RFV to determine whether the word becomes a key in the index. The choice of 14 for the purpose of computing the number of word repetitions allows the entire repetition count vector to occupy only 256 megabytes. The repetition count vector is discarded as soon as the RFV is initialized, with the latter occupying even smaller space.

The next step is the indexing of cDNA and genomic sequences. The sequences are concatenated and encoded using two bits per residue. The cDNA sequences are indexed first, with each word checked against RFV using the procedure described above. If the check is successful, the underlying 32-bit value (key) is written alongside its global coordinate. Such key/position pairs are accumulated unless indexing another sequence would top a pre-defined maximum index volume size. At that point the pairs are sorted by keys and the index volume is saved on the disk. The indexing continues until all sequences have been parsed.

On the disk, the index is stored in two components. A position component lists global coordinates of the keys while the key component lists keys and offsets corresponding to them in the position component. Similar index representations have been used in a number of other applications [2,10].

Different filtering vectors are used during the indexing of the cDNA and the genomic sequences. RFV derived from the genome is applied at the cDNA indexing to filter out words that are over-represented in the genome. Similarly, a participation vector (PV), which is a bit vector with bits indicating presence of the keys in the cDNA index, is applied at the indexing of the genome. The vector occupies 512 megabytes in memory and is used to admit into the genomic index only those keys that are found in the cDNA index. Genomic words are extracted at every other position of the genome. Combined with the continuous sampling of cDNA sequences, this assures that every pair of perfectly matching sequence segments of length seventeen or longer will be found.

As both indices have been created, word matching is done very quickly through comparison of the key components of the indices. Indeed, since the key components are ordered by keys, finding each matching pair of keys is achieved by synchronous scanning of the components. For the same reason, the number of words corresponding to a matching key on the cDNA and the genome is found immediately. Matching words are recorded as pairs of global coordinates for every pair of the cDNA and genomic index volumes. Because of the way the index volumes are constructed (above), matching words for every pair of cDNA and genomic sequences are guaranteed to be confined to a unique pair of index volumes. This is essential for the compartmentization which must have all alignments between a pair of sequences available by the time it starts processing the pair. The matching words are merged along common diagonals and extended using the drop-off approach [11].

We refer below to our elementary matching algorithm as *Compart matching*, because the implementation of the algorithm is embedded in the same software tool that does the compartmentization. In the rest of this sub-section we evaluate the repeat filtering step, the effect of using the PV in Compart matching, and compare the results of the compartmentization based on local alignments computed with different methods.

Filtering of repeated DNA sequences has been a subject of much previous research. The most widely used repeat masking tool, RepeatMasker [12], relies on an external database of repetitive elements. A more recent tool, WindowMasker [13], masks repetitive DNA segments using only the genomic sequence itself. Having considered using either of these tools to mask genomic repeats, we eventually developed our own approach which proved to be better suited for the task of cDNA-to-genomic preliminary alignment. Our goal was to keep the level of repeat filtering moderate, because missing input local alignments can negatively impact accuracy of the compartmentization algorithm. We prefer to use the term *repeat filtering *when describing the algorithm in Compart because, unlike RM or WM, it effectively tags whole words rather than individual positions on the genome.

We evaluated the intensity of repeat filtering by comparing the number of genomic words kept out of the index. To do so, we disabled the PV and counted the number of words with their corresponding bit set in RVF. In sequences masked with RM or WM, only words with all residues masked were counted. According to our tests (Table 14), the repeat filtering in Compart resulted in significantly fewer words filtered out, the least number of words filtered out exclusively, and by far the best running time.

**Table 14:** Filtering of genomic repeats by different methods. *Q*_1 _is the fraction of masked words. *Q*_2 _is the fraction of words masked exclusively by the method. In case of RM and WM, a word was considered masked if all its bases were masked. The timing in each test was obtained using a single instance running on Intel Xeon 2.33 GHz/8 GB Linux box.

		RepeatMasker	WindowMasker	Compart
Human	*Q*_1_(%)	46.79	28.81	17.68
	*Q*_2_(%)	31.03	12.23	5.41
	time (min)	6077	131	7
Mouse	*Q*_1_(%)	40.55	28.61	17.71
	*Q*_2_(%)	25.24	12.41	5.46
	time (min)	5955	117	6

Fewer repeated words filtered out may result in explosive growth of alignments in a general-purpose local alignment algorithm. In Compart, however, only alignments composing compartments are kept beyond the compartmentization step which is a small fraction of the alignments generated internally.

The RFV is used at the indexing of the cDNA sequences, which also initializes the PV. Table 15 lists the size of the key component of the genomic index for human and mouse as a percentage of what that size would be if the PV was not used. A more compact genomic index has an impact on computing time. In our experiment where human mRNA sequences were aligned against the reference genome, the indexing (search) has slowed by a factor of three (eleven) when the PV was disabled. The bulk of search performance improvement comes from non-redundancy of the index components, which eliminates duplicate look-ups and dramatically improves CPU cache line coherence.

**Table 15:** Impact of the PV on the size of the index. *N *is the number of full-length mRNA sequences. *R *is the number of genomic index keys as a percentage of the number of distinct words on the unmasked genome.

		Human 36.3	Mouse 37.1
mRNA	*N*	214 749	240 299
	*R *(%)	8.4	10.2
EST	*N*	7 732 838	4 836 245
	*R *(%)	38.6	27.2

Table 16 reports a test where compartments were produced based on alignments computed with Compart and Megablast, with Megablast using the genomic sequence masked with RepeatMasker or WindowMasker. To have Megablast account for the masking, the genomic sequence was used as the query. The option allowing Megablast to extent alignments over masked regions has been selected. All other Megablast options, including the word size of 28, have been set to their defaults. The table presents various mRNA sequence counts and the time spent to compute local alignments and compartments. Use of Compart resulted in more sequences in each category. It could have been possible to improve sensitivity of Compart even further by performing the extension of elementary matches along adjacent diagonals, but we have not done so. Note that local alignments for some mRNA sequences do not form compartments with any of the genomic sequences, which explains why the number of aligned mRNA sequences (*N*_1 _and *N*_2 _in the table) is always smaller than the number of sequences with at least one compartment (*N*_3_).

**Table 16:** Compartmentization based on local alignments computed using different methods. *N*_1 _is the number of mRNA sequences with 75% or higher coverage by alignments to any single chromosome or unplaced scaffold.*N*_2 _is the number of mRNA sequences with 75% or higher coverage by high-identity alignments to any single chromosome or unplaced scaffold. *N*_3 _is the number of sequences for which at least one compartment was identified with the minimum compartment identity of 75%. The computing time was collected on Intel Xeon 2.33 GHz/8 GB Linux box.

		MB/RM	MB/WM	Compart
Human 36.3	*N*_1_	232342 (94.95%)	234565 (95.86%)	238851 (97.61%)
	*N*_2_	224054 (91.56%)	226293 (92.48%)	229101 (93.63%)
	*N*_3_	231939 (94.79%)	234207 (95.71%)	236928 (96.82%)
	time (min)	179	70	32
Mouse 37.1	*N*_1_	227831 (98.44%)	228681 (95.17%)	232245 (96.65%)
	*N*_2_	222746 (92.70%)	223704 (93.09%)	226107 (94.09%)
	*N*_3_	226964 (94.45%)	227895 (94.84%)	230478 (95.91%)
	time (min)	372	59	29

A shortcoming of Compart matching algorithm is its reliance on perfectly matching keys. As sequences get more diverged (e.g. in cross-species alignments) the algorithm becomes less sensitive. In those cases, tools not relying on perfectly matching words, such as Megablast in discontiguous mode, will tend to provide better input for the compartmentization step.

### Compartmentization

For many cDNA sequences, their local alignments against the genome suggest more than one place from which these sequences (or their orthologous counterparts, in case of cross-species alignments) might have originated. The goal of the compartmentization step is to filter and partition the local alignments into subsets so that these subsets will pinpoint every candidate location on the genome. We use the term *compartment *to designate both the alignment subsets and the genomic locations. After a compartment is identified, a spliced alignment algorithm can step in to produce a more accurate alignment of the cDNA with the local genomic interval.

Compartments located on different chromosomes or different strands are trivially separated. For others, the task can be more complex because of possible sequencing errors, polymorphic sites and exon duplications. Various approaches have been employed in other tools to identify candidate genomic locations. In Spidey, a greedy algorithm is used in which high-stringency Blast hits are sorted by score and then iterated, possibly more than once. On every iteration, each hit is either skipped or assigned to its genomic window, based on whether the hit's coordinates are linearly consistent with those of the other hits already in the window. GMAP scans the ends of a cDNA in an attempt to find pairs of highly-specific oligomers matching into approximately the same location on the genome. The latter is defined taking into the account factors such as the allowed genomic expansion for a given length of the cDNA sequence, concentration of matches and collinearity of cDNA and genomic coordinates. SPA relies on BLAT to perform the compartmentization step, however the relevant algorithm is not described in the BLAT paper.

The compartmentization algorithm in Splign is based on a formally defined model of compartments. Consider a cDNA (*query*) sequence aligning in the sense direction with the plus strand of a genomic (*subject*) sequence. We call a *high-scoring pair *(HSP) a pair of intervals on the query and subject sequences revealing a certain level of similarity. Without a loss of generality, this exposition assumes that HSPs are ungapped and perfect.

Consider two HSPs, $h^{\left(k\right)}(l)=(h_{q}^{\left(k\right)}(l),h_{s}^{\left(k\right)}(l))$, 0 ≤ *l *≤ *L*_*k*_, where *L*_*k *_are the lengths of the HSPs, *k *= *i*, *j*. We introduce a binary relation over the set of HSPs to reflect the order in which exons or their parts follow. Say that *h*^(*i*) ^*precedes h*^(*j*) ^(*h*^(*i*) ^≺ *h*^(*j*)^) if the following conditions hold:

$$\begin{array}{l} h_{\alpha }^{\left(i\right)}(0)<h_{\alpha }^{\left(j\right)}(0)\\ h_{\alpha }^{\left(i\right)}(L_{i})<h_{\alpha }^{\left(j\right)}(L_{j}),\alpha \in \{q,s\}\\ h_{s}^{\left(j\right)}(l_{i,j}^{*})-h_{s}^{\left(i\right)}(L_{i})\leq I_{max} \end{array}$$

where *I*_*max *_is the upper limit on the length of introns and

$$l_{i,j}^{*}=\underset{\alpha \in \{q,s\}}{\max}\arg\underset{l\geq 0}{\min}\left|h_{\alpha }^{\left(j\right)}(l)-h_{\alpha }^{\left(i\right)}(L_{i})\right|$$

is a diagonal coordinate from which *h*^(*j*) ^may extend *h*^(*i*) ^as a part of the same or a different exon. The definition allows overlapping of HSPs and accounts for a possible deletion from the query which can be a result of evolution or an artefact. Such introduced binary relation implies a strict partial ordering over the full set *H *of HSPs.

For an arbitrary subset *C *= {*h*^(1)^,...,*h*^(*M*)^} of *H*, define its *query coverage *as the length of the part of the query covered by HSPs from C:

$$Q(C)=\left|\overset{M}{\underset{i=1}{\cup }}\left[h_{q}^{\left(i\right)}(0),h_{q}^{\left(i\right)}(L_{i})\right]\right|$$

Let's call *C *a *compartment *if the above binary relation renders on *C *the structure of a totally ordered set:

$$h^{\left(i_{1}\right)}≺h^{\left(i_{2}\right)}≺\ldots ≺h^{\left(i_{M}\right)}$$

The key to formalizing the compartmentization problem is an observation that a proper organization of HSPs into compartments {*C*_*i*_} will maximize the *cumulative *query coverage ∑*Q*(*C*_*i*_), provided that each compartment maintains some minimal level of query coverage: *Q*(*C*_*i*_) ≥ *Q*_*min*_. Indeed, biologically compartments represent gene copies with every copy delivering its portion of the query coverage. While some exons may diverge significantly enough to escape being detected by a local alignment tool of choice, it may still be possible to identify a compartment accurately as long as its alignment delivers the query coverage above the threshold. In practice, we select *Q*_*min *_as the minimum of some fraction of the query's length and a constant.

The following relation is introduced to reflect our model's assumption that no two (same-strand) compartments corresponding to a query can overlap on the genome:

$$h^{\left(i\right)}⊣h^{\left(j\right)}\Leftrightarrow h_{s}^{\left(i\right)}(L_{i})<h_{s}^{\left(j\right)}(0)$$

Call a sequence of HSPs *v *= {*h*^(1)^,...,*h*^(*M*)^} *valid *if for every *i *<*j *either *h*^(*i*) ^≺ *h*^(*j*)^, or *h*^(*i*) ^⊣ *h*^(*j*)^, or both are true. A valid sequence of HSPs can be viewed as a chain of non-overlapping compartments and assigned with a score:

$$S(v)=\sum_{i}-Q_{min}+Q(C_{i})$$

The optimization target is then defined as

$${\bar{S}}_{H}=\underset{v\in V_{H}}{\max}S(v)$$

where *V*_*H *_is the set of all valid sequences over *H*. Let order HSPs in *H *so that

$$h_{s}^{\left(1\right)}(L_{1})\leq h_{s}^{\left(2\right)}(L_{2})\leq \ldots \leq h_{s}^{\left(N\right)}(L_{N})$$

assuring that for every *h*^(*i*) ^and *h*^(*i*) ^such that *h*^(*i*) ^≺ *h*^(*j*) ^or *h*^(*i*) ^⊣ *h*^(*j*)^, *i *is less than *j*. Let $H_{k}={\left\{h^{\left(i\right)}\right\}}_{i=1}^{k},V_{H_{k}}$, is the set of valid sequences over *H*_*k *_and ${\bar{S}}_{H_{k}}$ is its best score. Then the dynamic programming algorithm is described by the following recurrences.

$$\begin{array}{c} {\bar{S}}_{H_{1}}=-Q_{min}+\left|h^{\left(1\right)}\right|\\ s_{1}={\bar{S}}_{H_{1}}\\ {\bar{S}}_{H_{k+1}}=\max({\bar{S}}_{H_{k}},s_{k+1}),k=\bar{2,N-1}\\ s_{k+1}=\max(-Q_{min}+\left|h^{\left(k+1\right)}\right|,S_{open}^{\left(k+1\right)},S_{ext}^{\left(k+1\right)}\\ S_{open}^{\left(k+1\right)}=-Q_{min}+\left|h^{\left(k+1\right)}\right|+\underset{i:h_{i}⊣h_{k+1}}{\max}s_{i}\\ S_{ext}^{\left(k+1\right)}=\underset{i:h^{\left(i\right)}≺h^{\left(k+1\right)}}{\max}(s_{i}+L_{k+1}-l_{i,k+1}^{*}) \end{array}$$

As the target is evaluated, backtracking is used to restore the compartments contributing to ${\bar{S}}_{H}$.

### Refined sequence alignment

Every compartment is further refined with a more accurate sequence alignment algorithm (SAA), which is a combination of the global and local alignment algorithms. The use of the compartment's local alignments is two-fold. First, they define an interval on the genomic sequence on which to perform the alignment. Second, some of the local alignments can be used to accelerate the algorithm by dividing its dynamic programming space. Note that one should be very conservative in choosing the alignments to be used as pivots for the SAA, in order to avoid forcing the final alignment through one of alternatives that were equally favorable during the compartmentization. In Splign, only high-identity diagonal alignments that provide a one-to-one mapping between the sequences are selected. The last condition is verified by checking for possible overlaps among all local alignments between the two sequences. Each pivotal alignment is trimmed at the ends to allow enough slack space for the SAA to locate proper splice sites.

In the areas between the pivotal alignments, the global alignment algorithm is applied. At the areas stretching to the borders of the compartment, we use a variant of the local alignment algorithm in which one of the alignment's ends is fixed at the pivot. In all cases, the following scoring scheme is used:

*V*_*ij *_= max {*G*_*ij*_, *E*_*ij*_, *F*_*ij*_, *S*_*ij*_}

*G*_*ij *_= *V*_*i*-1,*j*-1 _+ *W*_*diag*_(*i, j*)

*E*_*ij *_= *W*_*s *_+ max {*E*_*i*,*j*-1_, *V*_*i*,*j*-1 _+ *W*_*g*_}

*F*_*ij *_= *W*_*s *_+ max {*F*_*i*,*j*-1_, *V*_*i*,*j*-1 _+ *W*_*g*_}

$$S_{ij}=\underset{I\in [I_{min},I_{max}]}{\max}(V_{i,j-I}+W_{intr}(j-I,j))$$

where *W*_*diag*_(*i, j*) is the substitution score, *W*_*g *_and *W*_*s *_are the gap opening and extension scores, and *W*_*intr*_(*j *- *I, j*) is the score of the intron starting at genomic position *j *- *I *+ 1 and ending at *j*. Assuming only two types of introns, consensus and non-consensus, we denote below their scores as *W*_*c *_and *W*_*nc*_.

Scoring schemes with affine gap penalties have been used in many tools (e.g. [2,14,15]) and have the advantage that algorithms using them can run in time and space proportional to the product of the lengths of the sequences. An important question is the choice of scores, as any particular score assignment defines the algorithm's preferences in shaping various alignment details such splicing signals or micro-exons. Our approach to assigning the scores was to explicitly consider various types of alignment alternatives and subject the scores to conditions reflecting what is perceived as the most plausible choice in every alternative.

A list of such alternatives and their respective scoring conditions is given in Table 17. Every line in the table is a separate alternative with A and B being mutually exclusive choices. Provided that the scores satisfy the conditions specified, it is straightforward to verify the following statement. Let *m*_*A *_and *m*_*B *_be the number of mismatches in alignments *A *and *B*, and Δ is a constant. Then for every *m*_*A *_and *m*_*B *_such that *m*_*A *_- *m*_*B *_< Δ (*m*_*A *_- *m*_*B *_≥ Δ), *A *will score higher (lower) than *B*.

**Table 17:** Score selection. *W*_*m *_(*W*_*ms*_) is the score for matching (mismatching) bases. Other notations are given in the text.

Alignment A	Alignment B	Conditions
a consensus intron and no indels	a non-consensus intron and no indels	$\Delta_{1}-1<\frac{W_{c}-W_{nc}}{W_{m}-W_{ms}}<\Delta_{1}$
a consensus intron and an indel	a non-consensus intron and no indels	$\Delta_{2}+\frac{W_{m}}{W_{m}-W_{ms}}-1<\frac{W_{c}-W_{nc}+W_{g}+W_{s}}{W_{m}-W_{ms}}<\Delta_{2}$
two consensus introns and no indels	a non-consensus intron and no indels	$\Delta_{3}-1<\frac{2W_{c}-W_{nc}}{W_{m}-W_{ms}}<\Delta_{3}$
a consensus intron and no indels	two consensus introns and no indels	$\Delta_{4}-1<\frac{-W_{c}}{W_{m}-W_{ms}}<\Delta_{4}$
two consensus introns and no indels	a non-consensus intron and an indel	$\Delta_{5}-1<\frac{2W_{c}-W_{nc}-W_{g}-W_{s}}{W_{m}-W_{ms}}<\Delta_{5}-\frac{W_{m}}{W_{m}-W_{ms}}$
a consensus intron and no indels	two consensus introns and an indel	$\Delta_{6}+\frac{W_{m}}{W_{m}-W_{ms}}-1<\frac{W_{g}+W_{s}-W_{c}}{W_{m}-W_{ms}}<\Delta_{6}$

In addition to the conditions in Table 17, we required the scores to satisfy

*W*_*g*_, *W*_*s*_, *W*_*c*_, *W*_*nc *_< 0; *W*_*m *_> 0; *W*_*c *_> *W*_*nc*_

Since the termini are not fixed in the alignment, the following condition is used to control the minimum length of perfectly matching terminal exons $L_{min}^{\left(term\right)}$

$$W_{c}>-W_{m}\times L_{min}^{\left(term\right)}$$

Finally, the following condition was applied to improve consistency between the intron and gap scores:

|*W*_*nc *_- *W*_*g *_- *W*_*s *_× *I*_*min*_| → 0

This concludes the linear programming problem that we used to compute the scores. Since quality of EST sequences is generally lower than that of full-length mRNA sequences, Splign scores for EST alignments are computed using higher Δ constants. Using higher Δ constants means that the identity around splice sites must be higher for the algorithm to introduce less frequent alignment features such as non-consensus splices and micro-exons.

## Reviewers' comments

### Reviewer's report I

Dr Steven Salzberg, University of Maryland, College Park, MD, United States

This paper describes the program splign, which aligns spliced transcripts (ESTs and cDNAs) to genomic DNA. The program is very accurate and relatively fast, though not the fastest available. The authors' experiments show that for several large data sets, its performance (measured as bases aligned, or % of transcripts aligned correctly) is usually superior to several of the best alternative programs out there. Overall Splign appears to be a robust program with excellent accuracy, and a very useful "splice site aware" alignment algorithm. It is already widely used and will no doubt continue to be.

All my comments and suggestions have been addressed satisfactorily.

### Reviewer's report II

Dr Arcady Mushegian, Stowers Institute, Kansas City, KS, United States

"A small fraction of mRNA sequences are deposited to GenBank as complemented strand." – what is the evidence that this fraction is small, is it higher for EST projects than for full-length cDNA projects, and can the proportion of the wrong strands be estimated? On the other hand, what if both strands are transcribed, as recent Affymetrix studies seem to suggest – any evidence of this in the data, especially perhaps evidence of splicing in the non-protein-coding strand?

#### Author response

*Indeed, we did not investigate the precise number of such mRNA sequences and therefore shall remove 'small' from the sentence. It was clear though that such sequences do exist. Some of incorrectly oriented sequences can be detected by looking for alignments in the original direction having multiple non-consensus splices, that become all-consensus, high-identity alignments when reversed and complemented. We found 240 (0.1% of the total) sequences satisfying the above condition. The assertion that the presence of such sequences may bias the experiment is even more valid if their fraction is bigger*.

*The suggestion that both strands of an mRNA can be transcribed is very interesting and deserves a separate study. In our full set, we did notice nine mRNAs with perfect, complete and all-consensus alignment in both directions*.

"Compartment test" – Something is missing in the description of the assay: among 9383 mRNAs, how many corresponded to the known areas of segment duplications?

#### Author response

*It would have been possible to build a set of sequences for this test by collecting mRNAs aligning to known areas of segment duplications. In the test we chose a different approach, in which we collected all mRNAs that were likely to align (at least partially) at more than one place per subject per strand, based on their blast hit coverage*.

*The answer to the asked question will of course depend on a specific list of known segment duplications. For example, using a list of gene clusters available at NCBI, we extracted cluster gene-associated accessions and found that 3398 (36%) accessions from Subset 2 match the list. Using a list built with the compartmentization algorithm resulted in 8923 (95%) matching accessions. A third list, based on BLAT alignments, revealed 8215 (87%) accessions in common*.

### Reviewer's report III

Dr Andrey Mironov, Moscow State University, Moscow, Russian Federation

The paper addresses an important problem, spliced alignment of mRNAs and ESTs to genomic sequence. The problem is of special interest in context of splicing analysis. The existing algorithms of nucleotide spliced alignment are not fast and accurate enough. The authors present a new spliced alignment algorithm that involves indexing words in the genome and mRNAs, repeats filtering, comparison of the indexes and creating compartments, refinement using a dynamic programming procedure.

The "Subset 1" was created using a rather weak filter. Nevertheless this subset is noticeably smaller than the full set. What is the reason for this? What part of the filter provides the strongest reduction?

#### Author response

*The ORF-related restrictions accounted for about one third of the reduction. For each of the other excluded sequences at least one method reported more than one alignment with identity at or above the threshold*.

## Competing interests

The authors declare that they have no competing interests.

## Authors' contributions

DL conceived the project, YK, AS, TT and DL designed the algorithms and conceived the tests, YK implemented the algorithms and the complementary software, conducted the tests and wrote the paper. All authors have read and approved the final manuscript.
